# Differentiated thyroid cancer and adverse pregnancy outcomes: a propensity score-matched retrospective cohort study

**DOI:** 10.3389/fped.2024.1377061

**Published:** 2024-09-12

**Authors:** Xin Li, Fang Mei, Wu-Cai Xiao, Fan Zhang, Shanghang Zhang, Peng Fu, Jing Chen, Rui Shan, Bang-Kai Sun, Shi-Bing Song, Chunhui Yuan, Zheng Liu

**Affiliations:** ^1^ Department of General Surgery, Peking University Third Hospital, Beijing, China; ^2^ School of Basic Medical Sciences, Health Science Centre, Peking University, Beijing, China; ^3^ Department of Maternal and Child Health, School of Public Health, Health Science Centre, Peking University, Beijing, China; ^4^ Department of Ultrasound, Peking University Third Hospital, Beijing, China; ^5^ National Key Laboratory for Multimedia Information Processing, School of Electronics Engineering and Computer Science, Faculty of Information and Engineering Science, Peking University, Beijing, China; ^6^ Information Management and Big Data Center, Peking University Third Hospital, Beijing, China

**Keywords:** differentiated thyroid cancer, pregnancy outcomes, propensity score matching, cohort, real-world data

## Abstract

**Background:**

Differentiated thyroid cancer (DTC) has been increasingly common in women of reproductive age. However, the evidence remains mixed regarding the association of DTC with adverse pregnancy outcomes in pregnant women previously diagnosed with DTC.

**Methods:**

We conducted a retrospective cohort study in the Peking University Third Hospital in Beijing, China between January 2012 and December 2022. We included singleton-pregnancy women with a pre-pregnancy DTC managed by surgical treatment (after-surgery DTC) or active surveillance (under-surveillance DTC). To reduce the confounding effects, we adopted a propensity score to match the after-surgery and under-surveillance DTC groups with the non-DTC group, respectively, on age, parity, gravidity, pre-pregnancy weight, height, and Hashimoto's thyroiditis. We used conditional logistics regressions, separately for the after-surgery and under-surveillance DTC groups, to estimate the adjusted associations of DTC with both the composite of adverse pregnancy outcomes and the specific mother-, neonate-, and placenta-related pregnancy outcomes.

**Results:**

After the propensity-score matching, the DTC and non-DTC groups were comparable in the measured confounders. In the after-surgery DTC group (*n* = 204), the risk of the composite or specific adverse pregnancy outcomes was not significantly different from that of the matched, non-DTC groups (*n* = 816; *P* > 0.05), and the results showed no evidence of difference across different maternal thyroid dysfunctions, gestational thyrotropin levels, and other pre-specified subgroup variables. We observed broadly similar results in the under-surveillance DTC group (*n* = 37), except that the risk of preterm birth, preeclampsia, and delivering the low-birth-weight births was higher than that of the matched, non-DTC group [*n* = 148; OR (95% CI): 4.79 (1.31, 17.59); 4.00 (1.16, 13.82); 6.67 (1.59, 27.90)].

**Conclusions:**

DTC was not associated with adverse pregnancy outcomes in pregnant women previously treated for DTC. However, more evidence is urgently needed for pregnant women with under-surveillance DTC, which finding will be clinically significant in individualizing prenatal care.

## Introduction

Since the implementation of China's two-child policy (1), the proportion of high-risk pregnancies increased from 15.7% in 2008 to 24.7% in 2016 and exceeded 30.0% in 2018 (2). The corresponding increase in adverse pregnancy outcomes has brought new challenges to the reduction of maternal mortality ratio (3–5). Along with this, females have also faced the 2nd most commonly diagnosed cancer related to pregnancy: thyroid cancer. Of all thyroid cancers, differentiated thyroid cancer (DTC) accounts for more than ninety percent. Notably, the incidence of DTC shows an increasing trend among women of reproductive age (6). Recently, a focus on patients with a pre-pregnancy DTC has been strengthened in the Guidelines (2022) for Prevention and Management of Thyroid Diseases During Pregnancy and Perinatal Period in China (7).

Women with DTC are “candidates” for high-risk pregnancies. They would primarily undergo thyroidectomy surgery to manage DTC. They would also receive postoperative thyrotropin (TSH) suppression therapy (the thyroid hormone therapy used to suppress serum TSH levels lower than the normal range) to minimize the risk of DTC recurrence (8). Both surgery and postoperative hormone therapy may result in hypothyroidism or hyperthyroidism (9, 10), and this is more frequently occurring in pregnant women due to the fluctuation of gestational thyroid hormones. As an alternative to immediate surgery, active surveillance is a viable option for appropriately selected patients with low-risk DTC (11). Active surveillance refers to a period of close monitoring of cancer progression without performing immediate surgery. Compared to patients with immediate surgery, patients under active surveillance were shown to be at higher anxiety due to the risk for possible DTC progression (12, 13), predisposing them to adverse pregnancy outcomes.

It is important to clarify whether DTC is associated with the risk of adverse pregnancy outcomes. Notably, multiple risk factors other than DTC have been reported to relate to adverse pregnancy outcomes (14, 15). Theoretically, pregnant women would benefit from all prevention measures for adverse pregnancy outcomes. Practically, it is often not possible to implement such prevention measures at scale due to a lack of resources in real-world settings. To optimize resource allocation for prenatal care, pregnant women are often stratified into high-, moderate-, or low-risk groups and receive targeted interventions corresponding to their risk stratification. Therefore, if DTC is indeed associated with an elevated risk of adverse pregnancy outcomes, DTC should be considered as one risk factor in the management system of risk stratification for pregnant women; if not, clinicians can inform patients that additional prenatal examinations specific to DTC are unnecessary in most cases. This shared decision-making between clinicians and patients is not only helpful in relieving the current strain of medical resources but also in reducing the patients’ psychological burden due to the cancer “label” attached to them.

Controversy remains regarding the association of DTC with adverse pregnancy outcomes (16). This could be interpreted by the study population, controlled confounders, study outcomes, analysis methods, or effect modification. Until now, no studies on the present topic have focused on pregnant women with a pre-pregnancy DTC managed by active surveillance (briefly termed under-surveillance DTC below). To fill the research gap, our study included patients with under-surveillance or after-surgery DTC, adopted propensity-score matching (PSM) to control confounding effects (17) and clarified the association of DTC with the risk of adverse mother-, placental-, or neonatal-related pregnancy outcomes. We also conducted several subgroup analyses to examine the potential factors that modify the exposure-outcome associations. The findings of this study would have important significance for clinical decision-making in individualizing prenatal care.

## Methods

### Study population

Our multidisciplinary research team included experts in the fields of surgery, pathology, ultrasound, obstetrics, endocrinology, and maternal and child health. We conducted a retrospective cohort study at Peking University Third Hospital in Beijing, China between January 2012 and December 2022.

For the exposed group, we included singleton-pregnancy women with a pre-pregnancy DTC managed by active surveillance (under-surveillance DTC) or surgical treatment (after-surgery DTC). We excluded those with a diagnosis of malignant tumors other than DTC. If women had multiple pregnancies, we only included their first pregnancy. Specifically, women were eligible for the under-surveillance DTC group if they: (1) undergo no less than 2 rounds of examinations of neck ultrasonography during surveillance (before surgical treatment), (2) be under surveillance for at least 6 months, and (3) have no evidence of lymph node metastasis or extrathyroid invasion at baseline examination. Women were eligible for the after-surgery DTC group if they: (1) be treated with total thyroidectomy or lobectomy, and (2) have follow-up (after-surgery) examinations of neck ultrasonography or measurements of serum thyroglobulin (Tg) levels and Tg antibodies.

For the control group, we included singleton-pregnancy women without a pre-conception diagnosis of DTC and further obtained a propensity score (PS) matched population to balance measured confounders between the DTC and non-DTC control groups. We took two steps for the PS matching. First, we used logistic regression to estimate the PS (18), i.e., the probability of an individual included in the exposed group conditional on the observed confounders (*X*) at baseline: $e_{i}=P_{r}(Z_{i}=1|X_{i})$. Based on literature review (16, 19–25) as well as domain knowledge of the present topic, we selected observed confounders including age, parity, gravidity, pre-pregnancy weight, height, and Hashimoto's thyroiditis. Then, we performed the matching at a 1:4 ratio with a caliper of width equal to 0.4 standard deviation of the logit of the PS. This choice of caliper width was made after careful consideration of both the balance of baseline confounders and the sample size, building on our prior work (26, 27). Data were carefully collected from medical records by two researchers with rich experiences in clinical practice (X.L.) and statistical analyses (W.C.X.). This study obtained approval from the Medical Research Ethics Committee of Peking University Third Hospital (No. IRB00006761-M2022721).

### Pregnancy outcomes

Taking advantage of well-documented, fine-grained clinical records, we were able to collect a variety of mother-, neonate-, or placenta-related pregnancy outcomes. The primary outcome was the composite of adverse pregnancy outcomes (i.e., the occurrence of any adverse pregnancy outcomes). The secondary outcomes were the specific type of adverse pregnancy outcomes including mother-, neonate-, or placenta-related pregnancy outcomes. Mother-related pregnancy outcomes included assisted reproduction, cesarean delivery, preterm birth, gestational diabetes mellitus, preeclampsia, abortion, premature rupture of membranes, precipitate labor, postpartum anemia, and postpartum hemorrhage. Neonate-related pregnancy outcomes included stillbirth, macrosomia, low birth weight, and small for gestational age. Placenta-related pregnancy outcomes included placental abruption, abnormal aggressive placenta, placenta previa, retained placenta, and battledore placenta.

### Statistical analyses

To compare differences between groups, we used the *t*-test and Kruskal-Wallis rank sum test for normally and abnormally distributed continuous variables, respectively, and the Pearson chi-square test or Fisher exact probability test (when the number of patients ≤ 5) for categorical variables. After the establishment of the PS model, we compared differences in baseline characteristics between the DTC and PS-matched non-DTC groups by using weighted regression based on the matching weight; specifically, we compared means and prevalence of baseline measured characteristics of subjects via the recommended standardized differences for continuous [Formula (1)] and dichotomous [Formula (2)] variables, respectively (28). We used the threshold value of standardized differences higher than 10% to indicate a meaningful imbalance in baseline covariate distribution between the DTC and non-DTC groups. We used conditional logistics regression models, taking into account the matching properties of the data, to assess the associations of DTC with pregnancy outcomes, for the under-surveillance and after-surgery DTC patients, respectively.

(1) $$\;d=\frac{\left({\bar{x}}_{\mathrm{expose}}-{\bar{x}}_{\mathrm{control}}\right)}{\sqrt{\frac{s_{\mathrm{expose}}^{2}+s_{\mathrm{control}}^{2}}{2}}}$$

(2) $$d=\frac{\left({\hat{\;p}}_{\mathrm{expose}}-{\hat{\;p}}_{\mathrm{control}}\right)}{\sqrt{\frac{{\hat{\;p}}_{\mathrm{expose}}(1-{\hat{\;p}}_{\mathrm{expose}})+{\hat{\;p}}_{\mathrm{control}}(1-{\hat{\;p}}_{\mathrm{control}})}{2}}}$$

(${\bar{x}}_{\mathrm{expose}}$ and ${\bar{x}}_{\mathrm{control}}$ denote the sample mean of the covariate in exposed and control subjects, respectively; $s_{\mathrm{expose}}^{2}$ and $s_{\mathrm{control}}^{2}$ denote the sample variance of the covariate in exposed and control subjects, respectively; ${\hat{\;p}}_{\mathrm{expose}}$ and ${\hat{\;p}}_{\mathrm{control}}$ denote the prevalence of the dichotomous variable in exposed and control subjects, respectively).

We further conducted pre-specified subgroup analyses at two levels. First, in both the DTC and non-DTC groups, we explored whether the exposure-outcome associations differed by maternal advanced age (≥35, <35 years), primipara (yes, no), overweight or obesity (yes, no), assisted reproduction (yes, no), overt hyperthyroidism (yes, no), subclinical hyperthyroidism (yes, no), overt hypothyroidism (yes, no), subclinical hypothyroidism (yes, no), Hashimoto's thyroiditis (yes, no), TSH score (reflecting the average levels of TSH during gestation), and TSH instability (reflecting the degree of fluctuation of TSH during gestation). Second, specifically in the DTC group, we examined whether the risk of adverse pregnancy outcomes differed by tumor type (papillary thyroid carcinoma; follicular thyroid carcinoma), surgery type (total thyroidectomy; lobectomy), response to therapy (excellent; incomplete), TSH suppression therapy (over treatment; mild under treatment; under treatment), the time interval between surgical treatment and conception. Details about the subgroup variables and the cutoffs are shown in Supplementary Table S1. Statistical analyses were performed using R software version 4.3 and Stata software version 16.0. *P* values less than 0.05 were considered statistically significant.

## Results

### Baseline characteristics of the DTC and non-DTC groups

**Figure 1:**
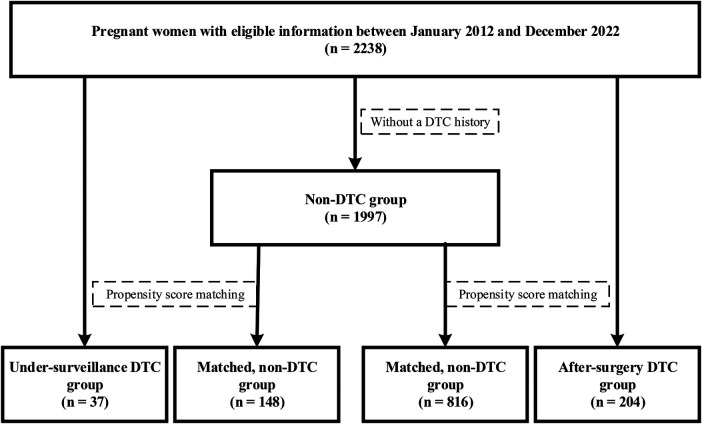
Flow chart of inclusion, exclusion, and PSM of the study participants.

**Figure 2:**
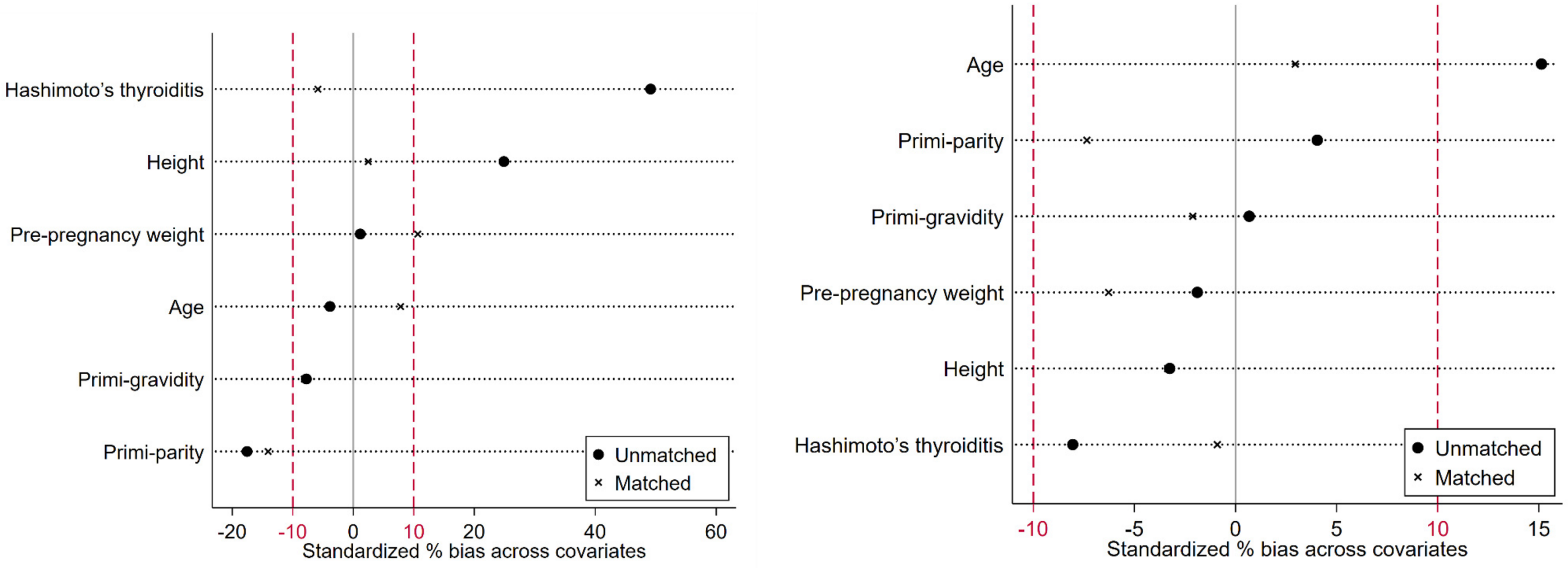
Standardized differences across the baseline measured confounders in the unmatched and matched comparisons (left: comparison between the under-surveillance DTC group and non-DTC group; right: comparison between the after-surgery DTC group and non-DTC group.).

The under-surveillance and after-surgery DTC groups included 37 and 204 pregnant women, respectively, and the correspondingly PS-matched, non-DTC control groups included 148 and 816 pregnant women, respectively (Figure 1). Before PS matching, the DTC and non-DTC groups differed in the distribution of maternal age and occurrence of Hashimoto's thyroiditis (Supplementary Table S2). After PS matching, the DTC group and the matched non-DTC groups were broadly similar in all measured confounders (Table 1; Figure 2).

**Table 1:** Comparison of baseline characteristics between the DTC and the matched, non-DTC groups.

Characteristics	Under-surveillance DTC group vs. non-DTC group			After-surgery DTC group vs. non-DTC group		
	Under-surveillance DTC group	Non-DTC group matched with under-surveillance DTC	*P*	After-surgery DTC group	Non-DTC group matched with after-surgery DTC	*P*
	(*n* = 37)	(*n* = 148)		(*n* = 204)	(*n* = 816)	
Age, year	33.16 ± 3.40	32.88 ± 3.68	0.733	33.91 ± 4.17	33.79 ± 4.02	0.770
Height, cm	163.12 ± 5.24	162.85 ± 5.51	0.829	162.07 ± 9.56	162.38 ± 9.77	0.749
Pre-pregnancy weight, kg	59.76 ± 6.70	57.90 ± 9.30	0.330	59.11 ± 11.14	60.27 ± 34.52	0.648
Gestational weight gain, kg	12.38 ± 4.67	11.93 ± 3.77	0.657	12.45 ± 4.50	11.86 ± 4.31	0.181
Primi-gravidity (%)	16 (43.24)	70 (47.30)	0.730	98 (48.04)	422 (51.72)	0.459
Primi-parity (%)	23 (62.16)	102 (68.92)	0.547	141 (69.12)	572 (70.10)	0.830
Hashimoto's thyroiditis (%)	17 (45.95)	72 (48.65)	0.819	39 (19.12)	159 (19.49)	0.952

In the under-surveillance DTC group, 91.7% and 8.3% of them were diagnosed with papillary thyroid cancer and follicular thyroid cancer, respectively; for the clinical TNM stage at the diagnosis, all were N0, and 73.0%, 21.6%, and 5.4% of them were T1a, T1b, and T2, respectively. In this study setting, no levothyroxine medication was administered to under-surveillance individuals to maintain low TSH levels. This was following the current Guidelines for the diagnosis and management of thyroid nodules and differentiated thyroid cancer in China (29).

In the after-surgery DTC group, 96.3% and 3.7% of them were diagnosed with papillary thyroid cancer and follicular thyroid cancer, respectively; for the pathological TNM stage, 58.1%, 30.2%, 3.5%, 3.5%, and 4.7% of them were T1a, T1b, T2, T3a, and T3b, respectively, and 79.8%, 14.6%, and 5.6% of them were N0, N1a, and N1b, respectively; for those with complete treatment information at follow-up (*n* = 70), 12.9% of them received the radioactive iodine (RAI) treatment.

### Pregnancy outcomes in the under-surveillance DTC group and PS-matched, non-DTC group

We compared the risk of adverse pregnancy outcomes between the under-surveillance DTC (*n* = 37) and PS-matched non-DTC (*n* = 148) groups in Table 2. There was no evidence of difference between the two groups in the composite of adverse pregnancy outcomes [OR (95% CI): 0.94 (0.33, 2.71); *P* = 0.913], mother- [OR (95% CI): 1.17 (0.42, 3.28); *P* = 0.762], neonate- [OR (95% CI): 2.06 (0.71, 6.01); *P* = 0.183], or placenta-related [OR (95% CI): 0.78 (0.21, 2,88); *P* = 0.708] adverse pregnancy outcomes. However, the under-surveillance DTC was associated with a higher risk of preterm birth [OR (95% CI): 4.79 (1.31, 17.59); *P* = 0.018], preeclampsia [OR (95% CI): 4.00 (1.16, 13.82); *P* = 0.028] and having a low-birth-weight baby [OR (95% CI): 6.67 (1.59, 27.90); *P* = 0.009], compared with the matched, non-DTC group.

**Table 2:** Associations of under-surveillance DTC, after-surgery DTC with adverse pregnancy outcomes.

Outcome indicators	Under-surveillance DTC group vs. non-DTC group				After-surgery DTC group vs. non-DTC group			
	Under-surveillance DTC group (*n* = 37)	PS matched, non-DTC group (*n* = 148)	OR (95% CI)	*P*	After-surgery DTC group (*n* = 204)	PS matched, non-DTC group (*n* = 816)	OR (95% CI)	*P*
Mother-related pregnancy outcomes	32 (86.49)	125 (84.46)	1.17 (0.42, 3.28)	0.762	183 (89.71)	702 (86.03)	1.43 (0.87, 2.35)	0.162
Assisted reproduction	7 (18.92)	14 (9.46)	2.36 (0.84, 6.63)	0.102	24 (11.76)	121 (14.83)	0.75 (0.46, 1.22)	0.243
Cesarean delivery	18 (48.65)	56 (37.84)	1.52 (0.75, 3.07)	0.245	75 (36.76)	299 (36.64)	1.01 (0.73, 1.39)	0.974
Preterm birth	6 (16.22)	7 (4.73)	4.79 (1.31, 17.59)	0.018	15 (7.35)	60 (7.35)	1.00 (0.55, 1.80)	1.000
Preeclampsia	5 (13.51)	5 (3.38)	4.00 (1.16, 13.82)	0.028	12 (5.88)	58 (7.11)	0.82 (0.43, 1.55)	0.536
Gestational diabetes	10 (27.03)	37 (25.00)	1.11 (0.50, 2.48)	0.803	56 (27.45)	226 (27.70)	0.99 (0.70, 1.39)	0.944
Abortion	2 (5.41)	2 (1.35)	4.00 (0.56, 28.40)	0.166	9 (4.41)	25 (3.06)	1.46 (0.67, 3.17)	0.341
Puerperal anemia	19 (51.35)	60 (40.54)	1.60 (0.75, 3.40)	0.222	82 (40.20)	343 (42.03)	0.93 (0.68, 1.26)	0.638
Premature rupture of membranes	3 (8.11)	28 (18.92)	0.38 (0.11, 1.33)	0.129	43 (21.08)	170 (20.83)	1.02 (0.70, 1.48)	0.938
Precipitate labour	0 (0.00)	2 (1.35)			1 (0.49)	6 (0.74)	0.67 (0.08, 5.54)	0.707
Postpartum hemorrhage	3 (8.11)	22 (14.86)	0.50 (0.14, 1.79)	0.289	29 (14.22)	121 (14.83)	0.95 (0.62, 1.47)	0.826
Neonate-related pregnancy outcomes	6 (16.22)	13 (8.78)	2.06 (0.71, 6.01)	0.183	32 (15.69)	117 (14.34)	1.11 (0.73, 1.69)	0.630
Stillbirth	0 (0.00)	1 (0.68)			0 (0.00)	0 (0.00)		
Macrosomia	1 (2.70)	4 (2.70)	1.00 (0.10, 10.07)	1.000	17 (8.33)	42 (5.15)	1.67 (0.93, 2.99)	0.085
Low birth weight	5 (13.51)	3 (2.03)	6.67 (1.59, 27.90)	0.009	11 (5.39)	45 (5.51)	0.98 (0.49, 1.95)	0.944
Small for gestational age	0 (0.00)	5 (3.38)	0.00 (0.00,.)	1.000	5 (2.45)	43 (5.27)	0.46 (0.18, 1.16)	0.100
Placenta-related pregnancy outcomes	3 (8.11)	15 (10.14)	0.78 (0.21, 2.88)	0.708	21 (10.29)	91 (11.15)	0.92 (0.56, 1.50)	0.729
Placental abruption	1 (2.70)	2 (1.35)			1 (0.49)	7 (0.86)	0.57 (0.07, 4.64)	0.601
Abnormal aggressive placenta	1 (2.70)	3 (2.03)	1.33 (0.14, 12.82)	0.803	5 (2.45)	17 (2.08)	1.18 (0.43, 3.19)	0.749
Placenta previa	0 (0.00)	0 (0.00)			1 (0.49)	0 (0.00)		
Retained placenta	0 (0.00)	2 (1.35)			3 (1.47)	31 (3.80)	0.38 (0.11, 1.25)	0.111
Battledore placenta	1 (3.23)	8 (5.93)	0.60 (0.06, 6.23)	0.672	12 (6.67)	39 (5.44)	1.30 (0.65, 2.61)	0.459
The composite of adverse pregnancy outcomes	32 (86.49)	129 (87.16)	0.94 (0.33, 2.71)	0.913	184 (90.20)	712 (87.25)	1.35 (0.81, 2.24)	0.249

### Pregnancy outcomes in the after-surgery DTC group and PS-matched, non-DTC group

We compared the risk of adverse pregnancy outcomes between the after-surgery DTC (*n* = 204) and PS-matched non-DTC (*n* = 816) groups in Table 2. There was no evidence of difference between the two groups in the composite of any adverse pregnancy outcomes (OR (95% CI): 1.35 (0.81, 2.24); *P* = 0.249), mother- (OR (95% CI): 1.43 (0.87, 2.35); *P* = 0.162), neonate- (OR (95% CI): 1.11 (0.73, 1.69); *P* = 0.630), or placenta-related OR (95% CI): 0.92 (0.56, 1.50); *P* = 0.729) adverse pregnancy outcomes.

### Subgroup analyses

**Figure 3:**
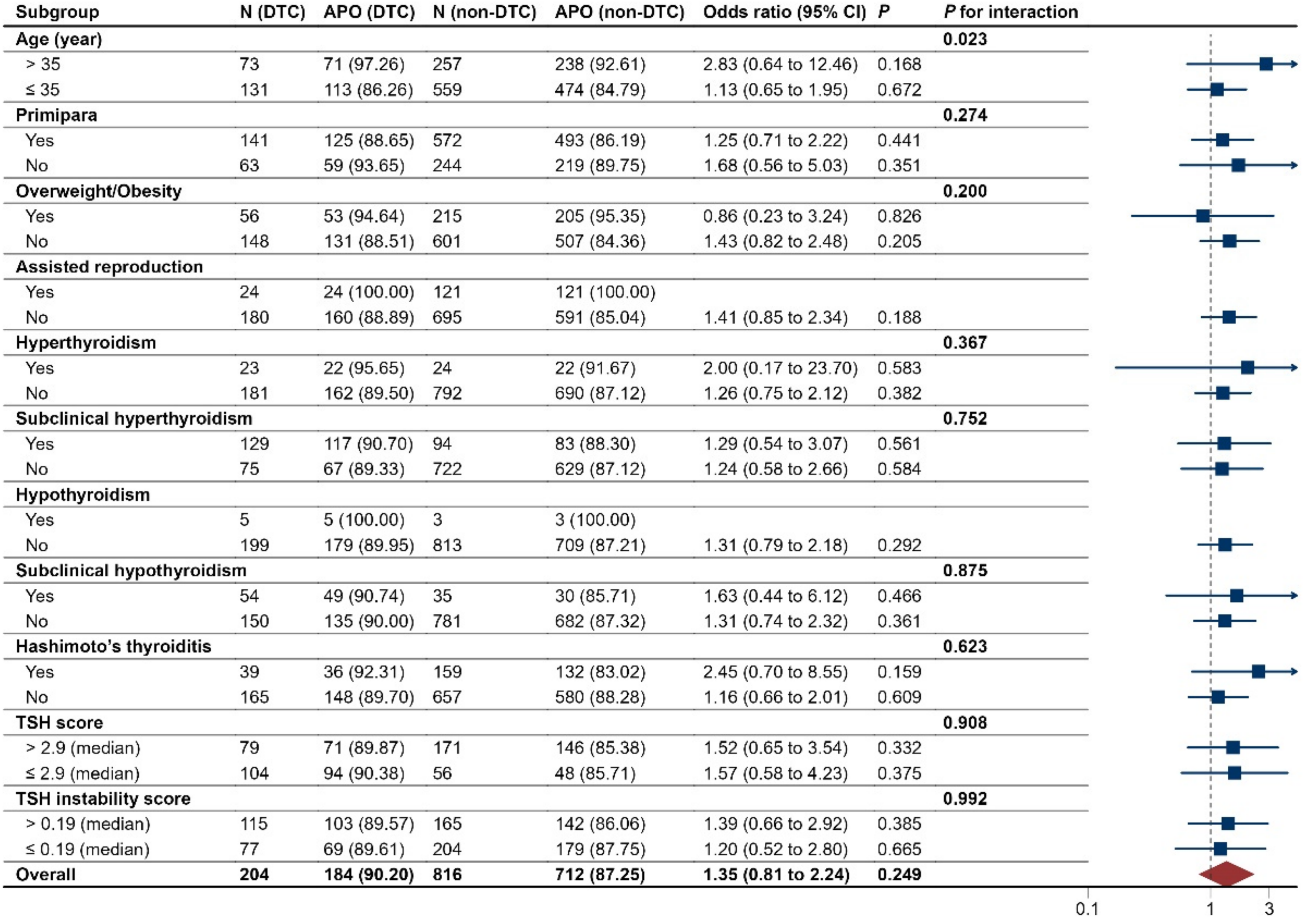
Subgroup analyses of pregnancy outcomes in the after-surgery DTC group and PS matched, non-DTC group. DTC, differentiated thyroid cancer; APO, adverse pregnancy outcomes. The median values of TSH in the after-surgery DTC group and PS matched, non-DTC group were 1.33 and 1.50 uIU/ml, respectively. The median values of FT4 in the two groups were 1.29 and 1.23 ng/dl, respectively.

The associations of after-surgery DTC with adverse pregnancy outcomes showed no differences across all prespecified subgroups (*P* for interaction > 0.05), except that the exposure-outcome association was stronger (*P* for interaction < 0.05) among those with maternal advanced age (≥35 years) than those with younger age (Figure 3).

Among pregnant women with after-surgery DTC, the risk of adverse pregnancy outcomes was shown no differences across tumor type, surgery type, response to therapy, TSH suppression therapy, and the time interval between surgical treatment and conception (all *P* > 0.05; Supplementary Figure S1).

## Discussion

This study was the first to examine the associations of DTC with adverse pregnancy outcomes, with a focus on both the under-surveillance and after-surgery patients. Taking advantage of the method of PS matching, we found that the DTC was not associated with the risk of adverse pregnancy outcomes in most cases, except for a higher risk of preterm birth, preeclampsia, and having a low-birth-weight baby in the under-surveillance DTC group.

The observed higher risk of some adverse pregnancy outcomes in the under-surveillance DTC group compared with that in the non-DTC group might be interpreted by patients’ poor psychological status during the surveillance period. For example, a prospective cohort study in China showed that patients with highly suspicious DTC had overall elevated anxiety and worse emotional function (13). Additionally, other studies indicated that higher levels of anxiety during pregnancy were associated with an increased risk of preterm birth (30), preeclampsia (31), and having a low-birth-weight baby (32). Thus, anxiety during pregnancy might bridge the association of under-surveillance DTC with some adverse pregnancy outcomes. Nevertheless, we should interpret this finding cautiously due to the modest sample size of patients with under-surveillance DTC in this study.

We did not observe evidence of differences in the risk of adverse pregnancy outcomes between the after-surgery DTC and non-DTC groups. This is potentially interpreted by China's highly effective and efficient management of high-risk pregnancies during prenatal care (33). Prenatal care provided for pregnant women is often more intensive in the study setting as a modernized and comprehensive upper first-class hospital in Beijing, China. However, we should note that patients with DTC and maternal advanced age were more susceptible to adverse pregnancy outcomes compared to those with younger age, as shown in our subgroup analysis results.

Our study contributes to the research field from at least two aspects. First, previous studies have been only focused on patients receiving surgical treatment for DTC but little attention has been paid to those receiving active surveillance, for the present topic (16). It should be noted that the underlying mechanisms for the association of DTC with pregnancy outcomes probably differ between individuals with surgical treatment and those with active surveillance. The former was more likely to experience thyroid dysfunction due to thyroidectomy or the postoperative TSH suppression treatment, while the latter was more likely to experience psychological burdens such as anxiety due to the risk of disease progression. We have preliminarily filled this research gap by conducting analyses separately for the two groups of individuals, and we indeed observed distinct findings between them.

Second, the associations of DTC with adverse pregnancy outcomes have been reported inconsistently across the previous studies (16, 19–25). One potential interpretation is that some factors might modify the exposure-outcome association. Taking advantage of our data with detailed clinical, surgical, pathological, and endocrinological records, we have conducted multiple subgroup analyses across patient-, disease-, treatment-, hormone-, and other variables based on the literature review, previous research work (26, 27), and domain knowledge of our multidisciplinary research team.

Our study had several limitations. The type of DTC included in this study was mainly papillary thyroid carcinoma and thus the finding may not be directly translated to other types of DTC. We are conducting a prospective study on other types of DTC including follicular, medullary, and poorly differentiated thyroid carcinoma and the findings will be reported in the future. Additionally, the study was based on a single institution in China, limiting the generalizability to other populations. Also, some results in this study might not have enough statistical power due to the moderate sample size and relatively low incidence of some adverse pregnancy outcomes.

Our study also had advantages. First, our study was based on well-documented, fine-grained clinical records, and before data analyses, all of the diagnosis and treatment information was carefully checked by the senior clinicians. This strict quality control for study data ensured the accuracy and reliability of our study findings. Second, we have used the PS matching method to obtain a matched comparison group and reduce the confounding bias in this observational cohort study, making our findings less likely to be biased (17). Third, we have conducted several subgroup analyses, findings of which could not only elucidate the robustness and consistency across subgroups but also clarify the potentially important modification effects such as the maternal advanced age.

Our study had important implications for future research. First, active surveillance has become gradually adopted worldwide to manage low-risk DTC in properly selected patients, in addition to immediate surgery (34–36). Our study is among the first attempts to explore the association of under-surveillance DTC with adverse pregnancy outcomes. We called for future studies to prospectively observe pregnant women with under-surveillance DTC to assess their risk of adverse pregnancy outcomes and validate the findings from our study. Further, it is important to carefully assess whether the DTC influences long-term outcomes for mothers and their offspring. Moreover, as the PS matching could not control the unmeasured confounders (17), it is important to collect baseline lifestyle factors in the exposed and controlled group before we can make a firm conclusion for the present topic.

Our study also had important significance for clinical practice. Our results suggested that women of reproductive age who have a pregnancy plan might, in most cases, not need to be very anxious about the elevated risk of adverse pregnancy outcomes. However, more intensive prenatal care might be considered to provide for patients with DTC who were receiving active surveillance (13) and those with maternal advanced age.

## Conclusion

The risk of adverse pregnancy outcomes in women previously treated for DTC did not differ from that in the well-matched, non-DTC women. However, our findings highlight the need to closely monitor the risk of adverse pregnancy outcomes among DTC patients under active surveillance in future studies.

## Data Availability

The raw data supporting the conclusions of this article are available upon request from the corresponding author.
