# Supplementary material for: Differentiated thyroid cancer and adverse pregnancy outcomes: a propensity score-matched retrospective cohort study
Source: Front Pediatr. 2024 Sep 12;12:1377061. doi: 10.3389/fped.2024.1377061 (PMC11424406; doi:10.3389/fped.2024.1377061)
Supplement: Supplementary file 1 [file Datasheet1.pdf]

List of supplementary files

- **Supplementary Table 1** Definition, diagnostic criteria, and cutoff value of subgroup variables
- **Supplementary Table 2** Comparison of baseline characteristics between the DTC and the unmatched non-DTC groups
- **Supplementary Figure 1** Subgroup analyses of pregnancy outcomes in the after-surgery DTC group

Supplementary Table 1 Definition, diagnostic criteria, and cutoff value of subgroup variables

Subgroup variable	Definition/Diagnostic criteria	Cutoff value
Hyperthyroidism	TSH< lower limit of reference range (or <0.1 mU/ L in early pregnancy), FT4 or FT3> upper limit of reference range.	
Subclinical hyperthyroidism	TSH< lower limit of reference range (or <0.1 mU/L in early pregnancy), and FT4 and FT3 are normal.	
Hypothyroidism	TSH> upper limit of reference range (or 4.0mU/L in first trimester) and FT4 < lower limit of reference range.	
Subclinical hypothyroidism	TSH> upper limit of reference range (or 4.0 mU/ L in first trimester) and FT4 in normal range.	
Hashimoto's thyroiditis	A group of thyroid diseases of autoimmune origin characterized by endothyroid lymphocyte infiltration and the detection of multiple thyroid autoantibodies in the blood. The TPOAb or TgAb exceeds the upper limit of the reference range provided by the kit.	
TSH score	TSH levels over a period of time were calculated based on multiple TSH measurements during pregnancy and the time interval between measurements. The range is 1-4, with a higher value indicating a higher TSH level during this period.	2.9 (median)
TSH instability score	Reflects the fluctuation of TSH during pregnancy. The value is between 0 and 6. The higher the value, the greater the TSH fluctuation.	0.19 (median)
TSH suppression therapy		
Over treatment	TSH inhibition therapy is overdone, leaving it at very low levels.	TSH < 0.1 mU/L
Mild under treatment	TSH inhibition therapy is insufficient. TSH is maintained at a high level.	TSH < 10 mU/L and TSH > 4 mU/L
Under treatment	TSH deficiency inhibition therapy. TSH remains at a very high level.	TSH > 10 mU/L

Note: Computational formula:

1. TSH score

$$\sum_{1}^{n} \frac{TS_n + TS_{n+1}}{2} \times ID_n \Big/ \sum_{1}^{n} ID$$

ID_n is each interval (days) and TS_n and TS_{n+1} are the TSH scores from the beginning and end of each interval, respectively[1].

<u>2. TSH instability score</u>

$$\sqrt{\frac{1}{N}\sum_{i=1}^{N-1}\left(\frac{c_{i+1} - c_i}{t_{i+1} - t_i}\right)^2}$$

C_i (i=1,…, N) are the N measurements of TSH values and t_i (i=1,…,N) are the timestamps at which measurements were recorded, so that $t_{i+1}–t_i$ is the difference in time between two subsequent measurements[2].

References:

1. Ito Y, Miyauchi A, Fujishima M, Noda T, Sano T, Sasaki T, Kishi T, Nakamura T. Thyroid-Stimulating Hormone, Age, and Tumor Size are Risk Factors for Progression During Active Surveillance of Low-Risk Papillary Thyroid Microcarcinoma in Adults. World J Surg. 2023 Feb;47(2):392-401. doi: 10.1007/s00268-022-06770-z. Epub 2022 Oct 2. Erratum in: World J Surg. 2022 Dec 13;: PMID: 36182976; PMCID: PMC9803751.

2. Taquet M, Griffiths K, Palmer EOC, Ker S, Liman C, Wee SN, Kollins SH, Patel R. Early trajectory of clinical global impression as a transdiagnostic predictor of psychiatric hospitalisation: a retrospective cohort study. Lancet Psychiatry. 2023 May;10(5):334-341. doi: 10.1016/S2215-0366(23)00066-4. Epub 2023 Mar 23. PMID: 36966787.

Supplementary Table 2 Comparison of baseline characteristics between the DTC and the unmatched non-DTC groups

	Under-surveillance DTC group (n = 37)	After-surgery DTC group (n = 204)	Unmatched non-DTC group (n = 1997)	***P*** *	***P*** #
**Age, year**	33.16 ± 3.40	33.91 ± 4.17	33.30 ± 3.88	0.826	0.034
**Height, cm**	163.12 ± 5.24	162.07 ± 9.56	162.37 ± 8.66	0.050	0.644
**Pre-pregnancy weight, kg**	59.76 ± 6.70	59.11 ± 11.14	59.46 ± 23.67	0.958	0.835
**Gestational weight gain, kg**	12.38 ± 4.67	12.45 ± 4.50	12.05 ± 4.38	0.698	0.218
**Primi-gravidity (%)**	16 (43.24)	98 (48.04)	919 (46.02)	0.635	0.582
**Primi-parity (%)**	23 (62.16)	141 (69.12)	1374 (68.80)	0.268	0.926
**Hashimoto's thyroiditis (%)**	17 (45.95)	39 (19.12)	447 (22.38)	0.001	0.284

* Comparison between the under-surveillance DTC group and the unmatched non-DTC group.

# Comparison between the after-surgery DTC group and the unmatched non-DTC group.

Variables	No.	Adverse pregnancy outcomes	Odds ratio (95% CI)	*P*
**Tumor type**				
FTC	3	3 (100.00)	-	
PTC	78	67 (85.90)	1.00 (1.00 to 1.00)	
**Surgery type**				
Lobectomy	39	35 (89.74)	-	
Total thyroidectomy	30	26 (86.67)	0.73 (0.16 to 3.44)	0.694
**Response to therapy**				
Incomplete	176	158 (89.77)	-	
Excellent	28	26 (92.86)	1.58 (0.31 to 8.05)	0.583
**TSH suppression treatment**				
Normal	194	175 (90.21)	-	
Over treatment	0	0 (.)	1.00 (1.00 to 1.00)	
Mild under treatment	5	5 (100.00)	1.00 (1.00 to 1.00)	
Under treatment	4	4 (100.00)	1.00 (1.00 to 1.00)	
**Time interval between pregnancy and surgery**				
< 2 years	27	23 (85.19)	-	
≥ 2 years	62	55 (88.71)	1.04 (0.25 to 4.39)	0.953

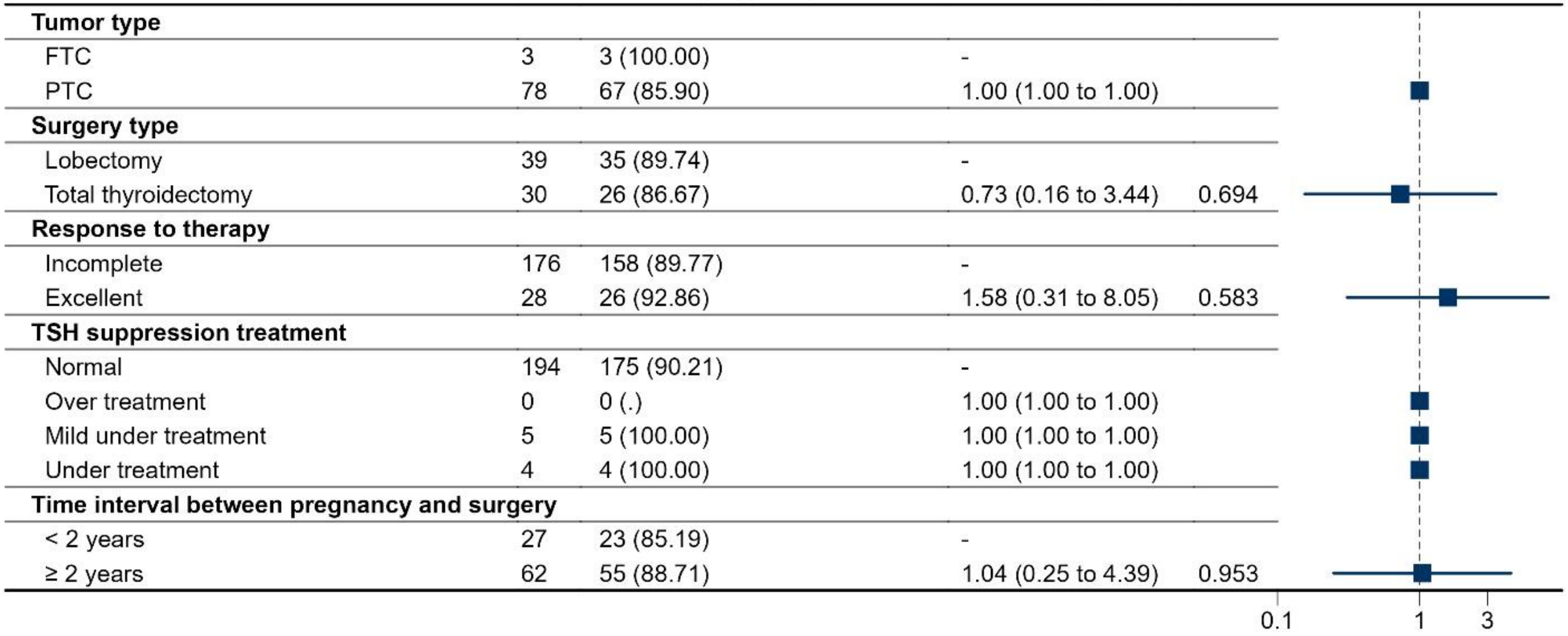

Supplementary Figure 1 Subgroup analyses of pregnancy outcomes in the after-surgery DTC group
